# Magnetic resonance-guided focused ultrasound for mesial temporal lobe epilepsy: a case report

**DOI:** 10.1186/s12883-020-01744-x

**Published:** 2020-04-29

**Authors:** Keiichi Abe, Toshio Yamaguchi, Hiroki Hori, Masatake Sumi, Shiro Horisawa, Takaomi Taira, Tomokatsu Hori

**Affiliations:** 1 Department of Neurosurgery, Tokyo Women’s Medical University, Shinjuku-ku, Kawata-cho, 8-1, Tokyo, 162-0054 Japan; 2 Department of Radiology, Shinyurigaoka General Hospital, Kawasaki, Japan; 3 Faculty of Advanced Techno-Surgery, Institute of Advanced Biomedical Engineering & Science, Graduate School of Medicine, Tokyo Women’s Medical University, Tokyo, Japan; 4 Department of Neurosurgery, Moriyama Neurological Center Hospital, Tokyo, Japan

**Keywords:** Focused ultrasound, Magnetic resonance guided, Mesial temporal lobe epilepsy, Centrality, Incident angle

## Abstract

**Background:**

We report the first case of transcranial magnetic resonance-guided focused ultrasound (MRgFUS) for mesial temporal lobe epilepsy (MTLE).

**Case presentation:**

The target was located 20 mm lateral from the midline and 15 mm above the skull base (left hippocampus). Despite the application of maximal energy, the ablation temperature did not exceed 50 °C, probably because of the low number of effective transducer elements with incident angles below 25 degrees. The skull density ratio was 0.56. Post-operative magnetic resonance imaging did not reveal any lesion and the patient remained almost seizure-free for up to 12 months.

**Conclusions:**

This preliminary case report suggests that MRgFUS may be effective for treating cases of MTLE. Therefore, the safety and feasibility of MRgFUS should be evaluated in future studies with larger numbers of participants and longer follow-up duration.

## Background

Mesial temporal lobe epilepsy (MTLE) is defined as partial epilepsy with the epileptogenic focus in the medial aspect of the temporal lobe. The seizure symptoms in MTLE are generally consistent with characteristic complex partial seizures (i.e., auras) and are accompanied by prodromal symptoms, including abdominal discomfort, tonic seizures, or automatisms. This type of epilepsy is extremely resistant to pharmacotherapy; however, when the epileptic focus is limited to one side, patients often experience good outcomes after surgical resection [1].

Previous studies have reported superior outcomes in MTLE following temporal lobectomy compared to treatment with anti-epileptic medications. In one randomised trial, patients who underwent temporal lobectomy experienced 58% improvement in seizures, compared to just 8% improvement in those who received medicines (*P* < 0.001) [1]. Additionally, minimally invasive treatments, including high-frequency (radiofrequency [RF]) coagulation surgery, stereotactic radiation therapy, and laser ablation (i.e., laser interstitial thermal therapy [LITT]), have been recently described for the treatment of MTLE [1–5]. However, complications, including visual-field defects, have been observed commonly following high-frequency coagulation surgery, LITT, stereotactic radiation therapy, and temporal lobectomy. Additionally, stereotactic radiation therapy has been associated with delayed-onset brain oedema and language memory defects, and these adverse effects influence patients’ decision to undergo surgical treatments [6–8].

Rapid progress has been made in the field of functional neurosurgery using transcranial magnetic resonance-guided focused ultrasound (MRgFUS) [9–11]. Additionally, reports have indicated successful use of MRgFUS for the treatment of essential tremor, Parkinson’s disease, obsessive compulsive disorder, and neuropathic pain [12–18]. We successfully performed the first MRgFUS treatment in Japan for a patient with medically refractory epilepsy.

MRgFUS ablation is performed with the ExAblate Neuro from Insightec (Haifa, Israel), which uses a 1024-element, 650 kHz phased array transducer to deliver the ultrasound energy and thermally ablate a focus that is located deep within the brain. Following a gradual increase in energy and temperature, a permanent lesion is created at the targeted location. The resulting thermal spot is monitored with real-time MR thermometry. However, studies have reported that the mesial temporal lobe is beyond the viable therapeutic range of the apparatus; therefore, a clinically appropriate increase in temperature cannot be achieved by MRgFUS in the target tissue. Potential limitations of MRgFUS ablation thus include the inability to attain desired ablation temperatures at deep targets, overheating of the skull, and other collateral effects.

The main objective of this report was to evaluate the safety and efficacy of MRgFUS for the treatment of MTLE. To our knowledge, this is the first report in the world of the use MRgFUS for treating MTLE. We also monitored for potential heating at the skull base region to identify potential safety issues.

## Case presentation

### Clinical history

A 36-year-old, right-handed woman presented to our clinic with complex partial seizures without automatism after experiencing nausea and auras (phantom visions). She was prescribed levetiracetam (1000 mg/day; 500 mg orally twice daily). While on the medication, she experienced seizures two to three times every month; however, off medication, she experienced seizures three to four times daily.

Before the patient underwent MRgFUS treatment, almost all of her epileptic attacks were complex partial seizures; however, she had experienced one episode of generalised seizure.

She had a normal birth and had not displayed any abnormalities in her developmental growth. There was no family history of epilepsy. At the age of 25 years, she developed epilepsy with partial seizures and prodromal symptoms, namely nausea and aura.

There were no abnormal neurological findings, and the scores of Verbal Intelligence Quotient 94, Performance Intelligence Quotient 99, and Full Intelligence Quotient 96 in WAIS-III before treatment were within the normal range.

Additionally, frontal lobe evaluation was performed; the patient’s Frontal Assessment Battery score was 17/18, and good frontal lobe functions were observed on the Wechsler Memory Scale—Revised as indicated by the following scores: verbal, 97; motility, 124; general, 105; attention, 5; concentration, 87; delayed reproduction, 99.

Pre-operative electroencephalogram findings were mostly normal, and only hypersensitivity due to light stimulation (≥3 Hz) was recognised. Epileptic seizures were characterised by déjà vu and gastric discomfort, with a few episodes of seizure per month before treatment during the course of oral levetiracetam administration. The seizures were symptoms of temporal lobe epilepsy, and based on positron emission tomography (PET) findings, a diagnosis of left temporal lobe epilepsy due to hypometabolism of the left temporal lobe was rendered. The patient sought surgical treatment to reduce her intake of epileptic drugs and because she had plans for pregnancy in future. She was reluctant to undergo selective hippocampectomy because of its association with a higher risk of cerebral dysfunction. She also declined the suggestion of gamma knife surgery because of the risk of cerebral oedema, a commonly reported side effect. Therefore, the patient underwent a left-sided hippocampal MRgFUS to treat her MTLE.

### Surgical procedure

The patient underwent pre-treatment magnetic resonance imaging (MRI) and computed tomography (CT). The MRI examination included high resolution T2-weighted fast spin echo scans of the sagittal, axial, and coronal planes, as well as routine sequences to evaluate the brain structures. Head CT images were used by the system to calculate the energy required by each transducer element for the ultrasound-guided transcranial penetration of the epileptic focus and for calculating the ratio between the bone and bone marrow of the skull (skull density ratio [SDR]). Fluorodeoxyglucose PET (FDG-PET) was performed to compare FDG metabolism before and after surgery.

**Fig. 1:**
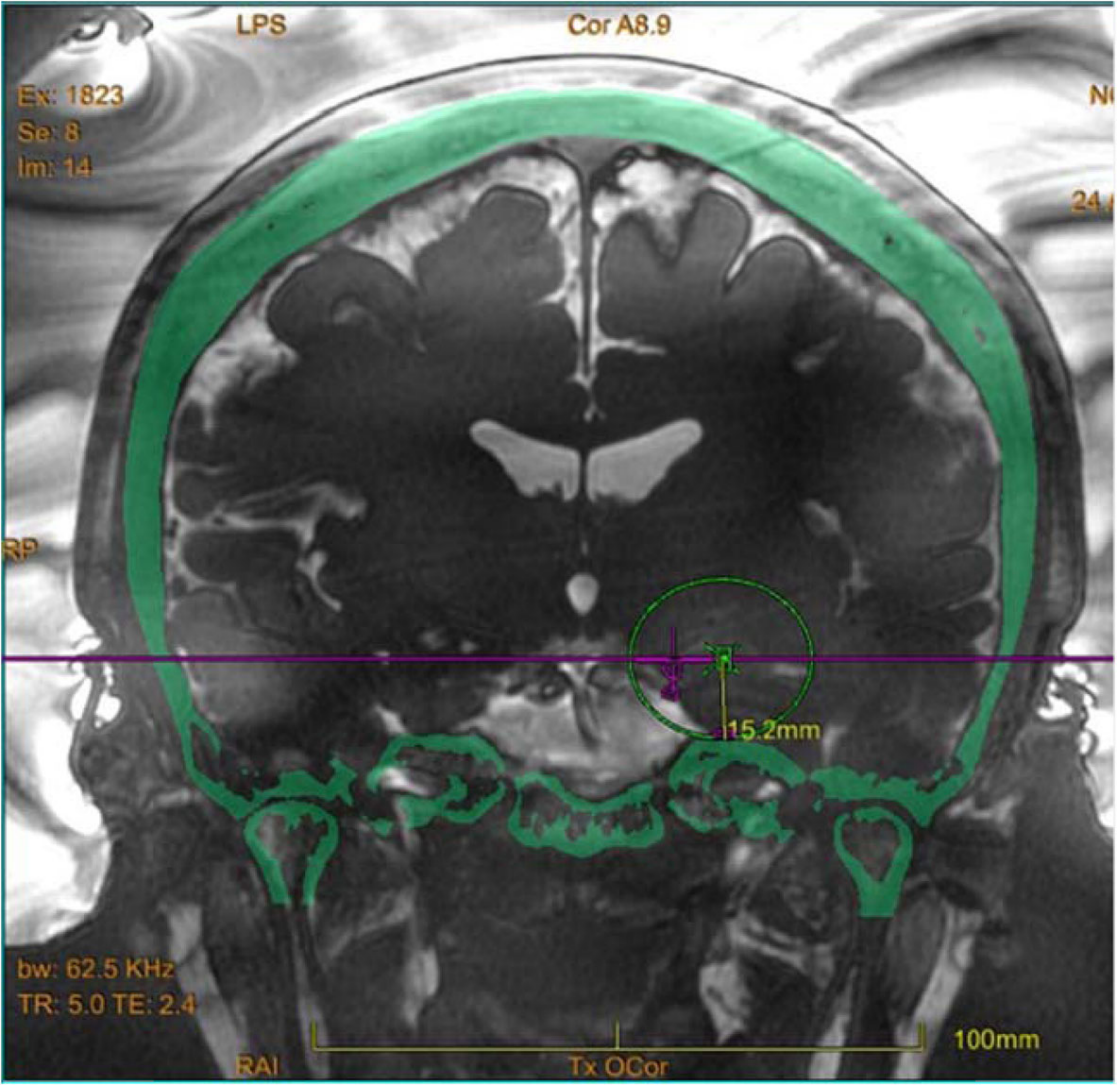
Live magnetic resonance imaging (MRI)

While under local anaesthesia, a Cosman-Roberts-Wells stereotactic frame was fixed onto the skull after shaving the patient’s entire scalp. We planned to perform multiple sonication sessions in the hippocampus. The first target was set at a distance of 15.2 mm above the skull base to avoid heating the semi-circular canal. In addition, the target was placed beyond the optic nerve and its temperature was monitored with MRI throughout the treatment (Fig. 1).

**Fig. 2:**
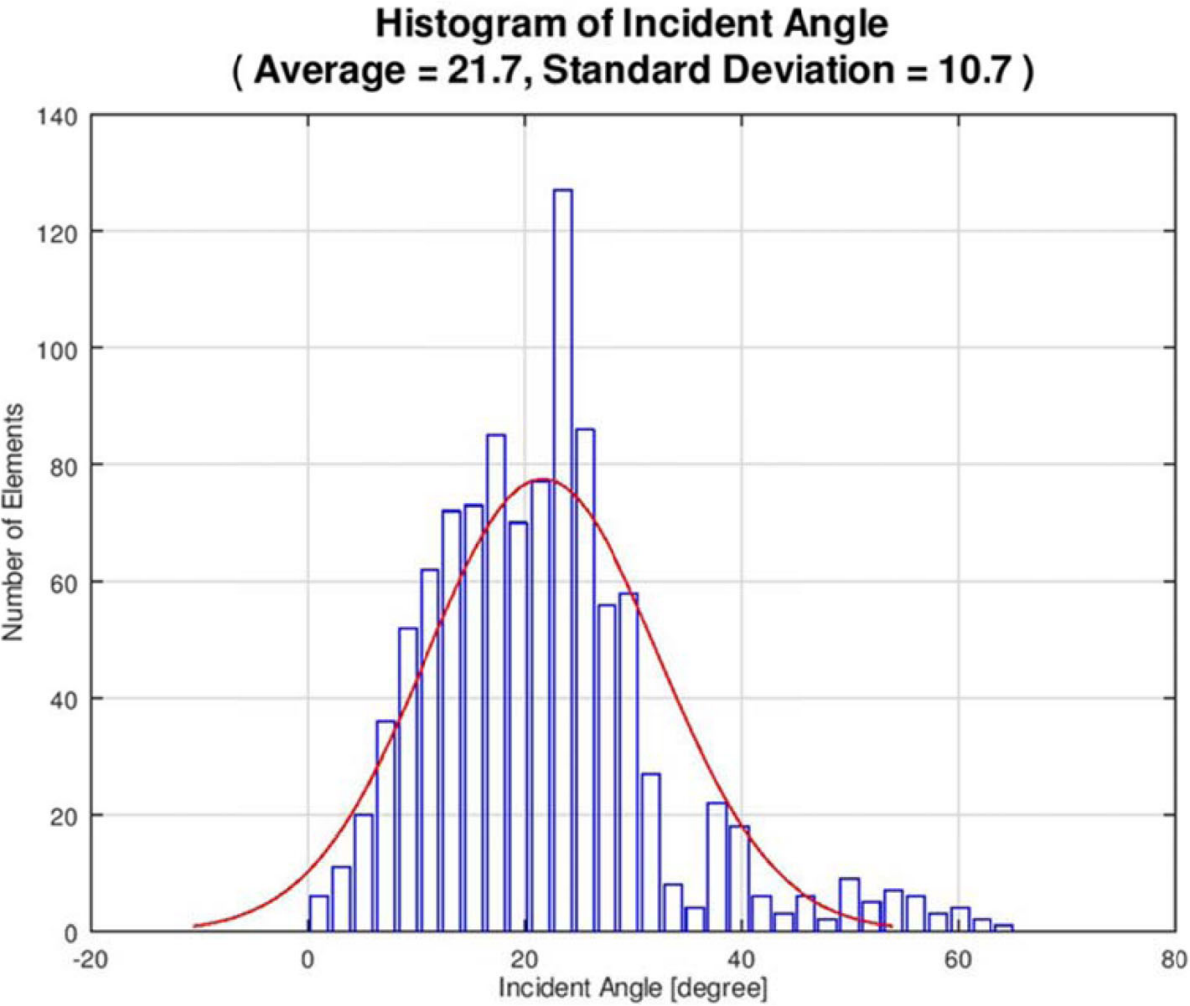
The incident angle histogram

**Fig. 3:**
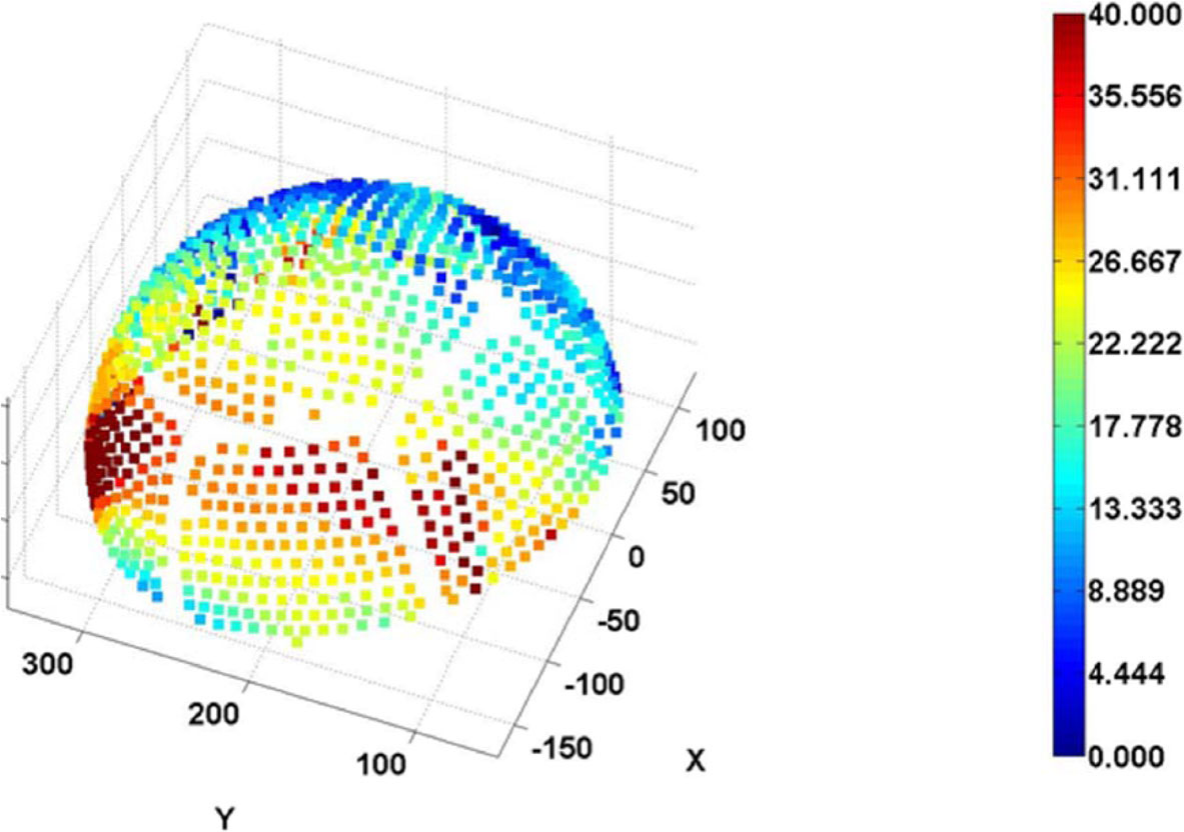
Incident angle distribution

**Fig. 4:**
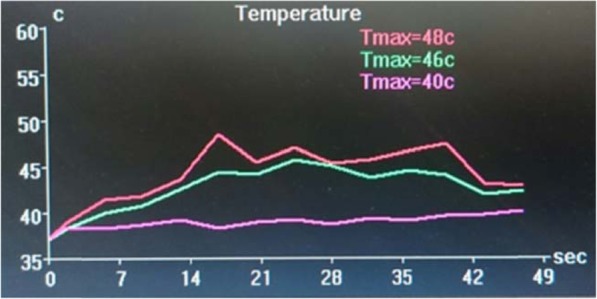
Brain temperature chart

The procedure was performed with a 3.0 Tesla MRI system (GE Healthcare, Chicago, Illinois) and ExAblate Neuro System (Insightec, Israel). Repetitive, low-power, 10–20-s long sonication sessions were performed to induce a temperature between 42 °C and 44 °C to ablate the target location. The patient reported severe vertigo and headache during the procedure, and thus, anaesthesia was induced intraoperatively. Overall, 12 sonication sessions were applied; however, the desired temperature of ≥54 °C could not be attained in the hippocampus. Since the transducer elements play a major role in energy delivery, we believe that we were unable to attain the desired ablation temperature because of the relatively small number of elements (659 of the 1024 total elements) with incident angles ≤25° (Figs. 2 and 3). The final temperature of the target reached 48 °C, and the actual delivered energy was 20,757 J (Fig. 4). The treatment was terminated because the maximal permitted energy could not generate the desired ablative temperature (≥ 54 °C).

### Post-operative course

Clinical follow-up was performed immediately after the procedure, at 1 week, as well as 1, 3, 6, and 12 months after MRgFUS lesioning. We evaluated the change in Engel classification from baseline to post-operative 12 months. In addition, changes in the overall Engel classification [19] relative to the baseline were obtained at each follow-up time point. MRI evaluations were performed immediately and at 1 month after the MRgFUS lesioning. There were no changes in MRI results at 1 month after the sonication sessions. Post-operative MRI showed no lesion or cerebral oedema at the targeted location.

One month after the treatment, the patient experienced a temporary worsening of seizure frequency (4 episodes of seizures per month during the menstrual cycle); however, her symptoms improved after the first month, and she remained almost seizure-free even at 12 months postoperatively despite consuming the same dose of levetiracetam (1000 mg/day) (Engel classification, class III). Although the patient’s seizure frequency improved, we did not observe a lesion on follow-up MRI at 1 month after the sonication sessions. Therefore, this finding will be evaluated by functional MRI and FDG-PET over a longer follow-up period.

**Fig. 5:**
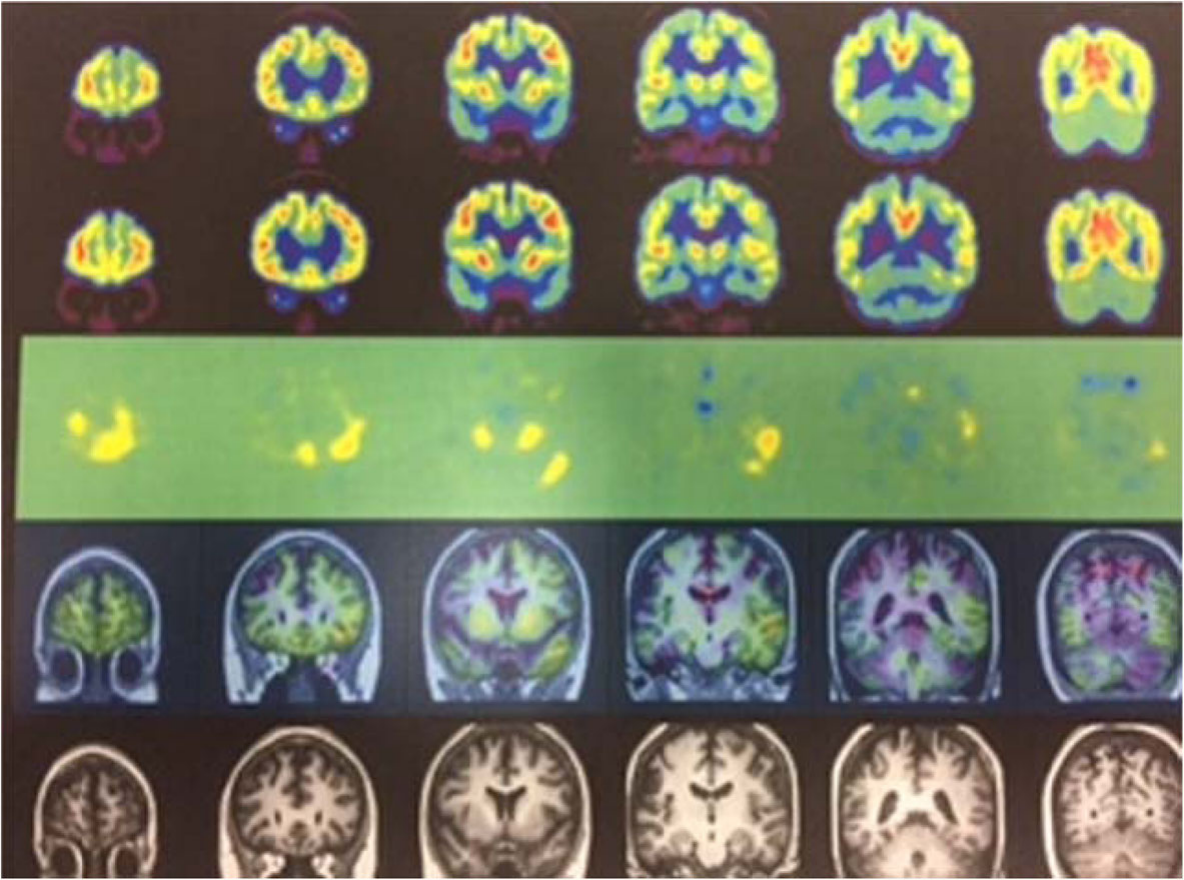
MRI-PET

Results of pre-operative FDG-PET demonstrated that the left medial temporal tip was hypometabolic. Differences between the pre-operative and post-operative FDG standardised uptake value ratios (SUVRs) revealed an increase in FDG metabolism in the left lateral temporal lobe, bilateral striata, bilateral frontal bases, and left posterior cingulate gyrus. Moreover, Statistical Parametric Mapping (SPM, Wellcome Trust Centre for Neuroimaging, London, UK) analysis showed a considerable decrease in the hypometabolic area postoperatively (Fig. 5).

At the 12-month follow-up visit, the patient was able to perform the activities of daily life with half the dose of levetiracetam (from 1000 mg/day to 500 mg/day). In addition, she experienced a healthy pregnancy one and a half years after treatment.

## Discussion and conclusions

The efficacy of surgical treatment for MTLE was established in the early twenty-first century. However, the effectiveness of minimally invasive treatments other than pharmacotherapy are still being explored. We used focused on the use of ultrasound (MRgFUS) for the surgical treatment of a patient with MTLE.

**Fig. 6:**
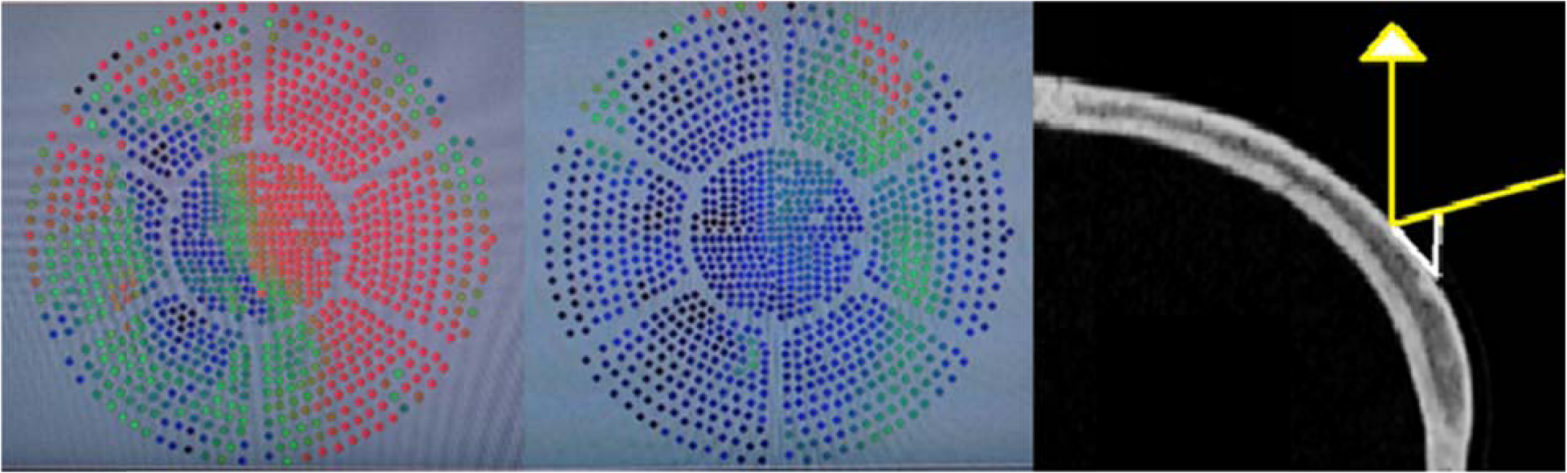
Comparison of the number of effective elements

When performing focused ultrasound ablation of the brain, the laterality of the target location within the skull should be considered for evaluation of the feasibility of epilepsy treatment. If the target is close to the centre of the brain (for example, the thalamic ventralis-oralis (Vo) nucleus for high-centrality focal hand dystonia), the ultrasonic incident angle decreases, and the permeability of ultrasound increases (Fig. 6). Although the SDR is an important factor for energy delivery through the bone, the importance of the incident angle is apparent from the present case. Furthermore, studies have reported that an incident angle of ≥25° decreases the permeability of ultrasound through the bone [20].

It has been reported that high-frequency coagulation is an effective and safe method for stereotactic coagulation of the hippocampus [3, 4]. Thus, our research question was to assess whether stereotactic coagulation of the hippocampus could be achieved with focused ultrasound.

Findings from previous studies have demonstrated that prolonged sonication time is required to perform irreversible coagulation in the medial temporal lobe, in addition to a simultaneous blocking algorithm [21]. Despite the long sonication time used in the current study, we were unable to irreversibly elevate the brain temperature of the targeted focus. Additionally, the patient described in this report experienced dizziness, most likely caused by heating of the skull base. However, this dizziness was reversible and only occurred during sonication performed in the awake state; therefore, we performed sonication under general anaesthesia after the patient complained of dizziness and headache. Additionally, we believe that we were unable to achieve irreversible heating in the hippocampus because the temperature was aligned to ensure reversible skull base heating. Therefore, it is necessary to investigate whether focused ultrasound of the hippocampus with irreversible skull base heating would be possible even if irreversible coagulation of the seizure foci is achieved in the hippocampus.

Overall, MRgFUS appears to be a safe procedure. Temporal lobe coagulation with gamma knife has been reported to cause delayed oedema and cyst formation; however, there have been no reports of delayed complications associated with focused ultrasound for treating essential tremor [7, 12, 18]. Similarly, studies on gamma knife treatment have reported that changes in MRI and PET findings appear 10 months after treatment [22]. Conversely, some studies have reported that patients with arteriovenous malformation (AVM)-related epilepsy experienced a reduction in epileptic symptoms without any changes in imaging findings [13]. In the current case, we could not achieve irreversible heating; therefore, there were no temporary oedematous changes, or other permanent changes on follow-up MRI. However, a follow-up PET scan revealed that metabolic changes occurred in the surrounding region rather than in the epileptogenic focus itself.

We were not able to demonstrate a coagulation focus in this patient with imaging. However, the clinical effects and PET-CT changes were apparent, and we considered two possibilities for this contradiction. One theory is that the underlying neuromodulatory effect due to MRgFUS has not yet been fully identified, although a putative mechanism of action has been reported [23]. The other possibility is that hippocampal vulnerability results in a change that cannot be visualised even at low temperatures due to the complicated cellular structure of the hippocampus [24]. One limitation of this work in that no follow-up pathological examination can be performed after ultrasonic sonication in living humans.

Studies have described the possible existence of neural networks in temporal lobe epilepsy [25]. Following MRgFUS, the improvements in epileptic symptoms as seen in our case may be due to improvements in these networks, vulnerability of the lesioned cells to relatively low temperatures, or seizure tract disruption [26].

Sub-ablation temperatures were achieved, and no discernible gliosis was detected on follow-up imaging examinations; nevertheless, the patient achieved significant resolution of seizures and epileptic symptoms. This could be a crucial finding, which suggests that focused ultrasound may induce neuronal changes that could either be less or different than those induced by typical ablation, but sufficient to possibly induce physiological changes to change regional seizure threshold. Moreover, this imaging observation shows that the focused ultrasound used for ablation in this study did not induce any lesions; however, the procedure was clearly efficacious, and demonstrable neuromodulation was observed, indicated by changes in FDG-PET findings.

In addition, the localization of the focused ultrasound and the accuracy of the target have already been mentioned, and in this case, it is considered that there was no region that unexpectedly or irreversibly affected the target focus [27].

This study has limitations. This is only the first case report. The follow-up period is also short.

Therefore more clinical studies are necessary. The patient was pregnant during the follow-up period. Since catamenial seizure frequency can change because of pregnancy, a longer follow-up period will better define the effects of sonication.

In conclusion, we provide promising evidence of the beneficial effect of MRgFUS for seizure control in a patient with MTLE. Further work in a larger group of patients is necessary to validate these findings and to define the optimal sonication parameters.

A longer follow-up duration is needed in future studies to validate the long-term absence of seizures.

## Data Availability

The datasets used and/or analysed during the current study are available from the corresponding author on reasonable request.

## Abbreviations

AVM: Arteriovenous malformation
CT: Computed tomography
FDG-PET: Fluorodeoxyglucose positron emission tomography
LITT: Laser interstitial thermal therapy
MRgFUS: Magnetic resonance-guided focused ultrasound
MRI: Magnetic resonance imaging
MTLE: Mesial temporal lobe epilepsy
RF: Radiofrequency
SDR: Skull density ratio
SPM: Statistical Parametric Mapping
SUVR: Standardised uptake value ratio
